# Energetic and reproductive costs of coral recovery in divergent bleaching responses

**DOI:** 10.1038/s41598-021-02807-w

**Published:** 2021-12-07

**Authors:** Sarah E. Leinbach, Kelly E. Speare, Ashley M. Rossin, Daniel M. Holstein, Marie E. Strader

**Affiliations:** 1 Department of Biological Sciences, Auburn University, Auburn, AL 36849 USA; 2 Department of Ecology, Evolution, and Marine Biology, University of California Santa Barbara, Santa Barbara, CA 93117 USA; 3 Department of Oceanography and Coastal Sciences, Louisiana State University, Baton Rouge, LA 70803 USA

**Keywords:** Marine biology, Climate-change ecology, Coral reefs

## Abstract

Mass thermal bleaching events are a primary threat to coral reefs, yet the sublethal impacts, particularly on energetics and reproduction, are poorly characterized. Given that the persistence of coral populations is contingent upon the reproduction of individuals that survive disturbances, there is an urgent need to understand the sublethal effects of bleaching on reproductive output to accurately predict coral recovery rates. In 2019, the French Polynesian island of Mo’orea experienced a severe mass bleaching event accompanied by widespread coral mortality. At the most heavily impacted sites, we observed *Acropora hyacinthus* individuals that were resistant to bleaching, alongside colonies that bleached but showed signs of symbiont recovery shortly after the bleaching event. We collected fragments from *A. hyacinthus* colonies five months post-bleaching and, using energetic assays and histological measurements, examined the physiological and reproductive consequences of these two distinct heat stress responses. Despite healthy appearances in both resistant and recovered corals, we found that recovered colonies had significantly reduced energy reserves compared to resistant colonies. In addition, we detected compound effects of stress on reproduction: recovered colonies displayed both a lower probability of containing gametes and lower fecundity per polyp. Our results indicate that bleaching inflicts an energetic constraint on the concurrent re-accumulation of energy reserves and development of reproductive material, with decreased reproductive potential of survivors possibly hampering overall reef resilience. These findings highlight the presence of intraspecific responses to bleaching and the importance of considering multiple trajectories for individual species when predicting population recovery following disturbance.

## Introduction

Coral reefs worldwide face unprecedented levels of stress caused by anthropogenic climate change. Elevated sea surface temperatures that trigger mass bleaching events are widely regarded as the greatest threat to coral reefs because they cause substantial coral mortality and threaten the persistence of corals as ecologically relevant framework builders^1,2^. Coral bleaching events are projected to increase in both frequency and severity in the near future^3^; hence, there is an urgent need to understand the consequences of recovery from climate change-induced temperature stress on coral physiology and reproduction in order to more accurately predict future population and community dynamics. Thermal stress is a major contributor to declines in coral cover^4^ and accordingly many studies on the impacts of coral bleaching have focused on mortality^5–7^. However, sublethal effects, particularly on reproduction, may play an important role in overall reef recovery following bleaching events because surviving colonies will populate the next generation of coral recruits^8,9^. Further, as the incidence of marine heat anomalies increases globally^3,10^, colonies that survive mass bleaching events may experience sublethal bleaching multiple times within their lifespan, warranting additional study on the sublethal impacts of bleaching on corals and reef resilience.

Reproduction, and ultimately fitness, is fundamentally influenced by the energetic condition of the coral holobiont^11,12^. The majority of corals’ daily energy requirements are met using photosynthetically fixed compounds translocated from their endosymbiotic microalgae^13^. During bleaching, this symbiotic relationship destabilizes, resulting in a considerable reduction in the amount of carbon provided to the host^14,15^. To compensate for the energetic deficit, corals must either increase heterotrophic feeding or catabolize stored energy reserves to meet their metabolic needs^16,17^. Under prolonged stress conditions, such as a severely bleached state, there are finite resources available that the coral must allocate to physiological processes such as tissue maintenance, defense, and reproduction^18^. Energy would likely be allocated towards one of these strategies that facilitate colony recovery (i.e., heterotrophic feeding or consuming energy reserves), rather than to non-essential life functions; this potentially limits energy diverted towards gamete production^19,20^.

Bleaching can induce profound negative effects on coral reproductive output, some of which may persist for multiple spawning seasons^21–23^. Bleaching events can lead to reductions in the percent of colonies that spawn within a population, and colonies that do spawn produce fewer gametes^21^. Heat stress has been linked to decreased energy reserves and consequently reduced fecundity and size of lipid-rich, energetically costly oocytes^24–26^. Colonies that undergo bleaching also display smaller spermary size and abundance and impaired sperm motility, although these negative effects on sperm persist for a shorter duration than those on oocytes^23,27^. However, the reproductive costs associated with coral bleaching are species-specific and related to the severity of the heat stress, highlighting the need for further investigation into reproductive output following bleaching across many species and locations^28–30^. What is not entirely reconciled is to what degree the bleaching response, as opposed to the heat stress itself, is responsible for these reproductive effects, and whether differential intraspecific bleaching responses have reproductive—and ultimately demographic—implications.

**Figure 1:**
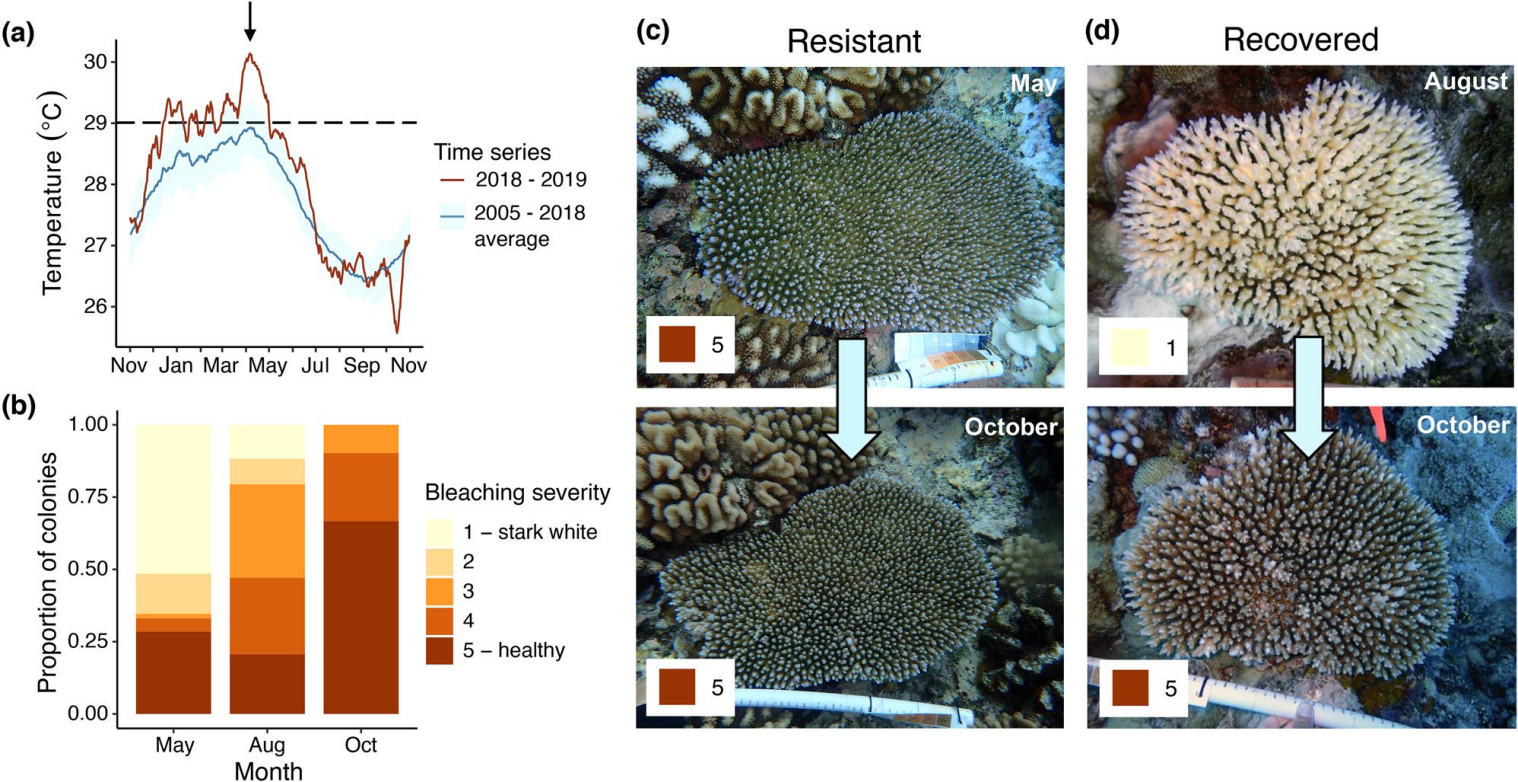
The 2019 bleaching event in Mo’orea, French Polynesia. (**a**) Average sea surface temperatures over 13 years (blue line) and observed sea surface temperature from November 2018–October 2019 (red line) at all LTER sites in Mo’orea. Dashed black line indicates the bleaching threshold for corals in Mo’orea. Heat anomaly and related bleaching event indicated with black arrow. (**b**) Proportion of surveyed colonies displaying various bleaching severities. Colonies were surveyed at the height of the bleaching event in May (N = 131), after three months of recovery in August (N = 34), and after five months of recovery in October (N = 51). (**c**) A colony resistant to bleaching photographed in May and October with associated bleaching scores. (**d**) A colony that bleached during the bleaching event in May and showed signs of recovery in August, and later recovered by October with associated bleaching scores.

Here, we examined the impact of thermal bleaching stress on stored energy reserves and reproductive output, two parameters which are critical for coral community recovery, in the tabular coral *Acropora hyacinthus,* one of the key reef-builders in the Indo-Pacific Ocean^31^. From December 2018 to May 2019, the island of Mo’orea, French Polynesia experienced a massive heat anomaly in which sea surface temperatures were sustained above 29 °C, the noted thermal stress accumulation threshold for corals in Mo’orea^32^, for a total of 115 days over a period of 139 days (Fig. 1a). The heatwave resulted in one of the most severe mass bleaching events ever recorded for the island. At the most highly impacted sites, > 80% of *Acropora spp.* colonies were bleached or dead in July 2019^33^. Despite widespread coral bleaching and mortality, recovery following the bleaching event was observed (Fig. 1b). There was also colony-level variability in the prevalence and severity of bleaching, including individuals that never showed any visual signs of bleaching (‘resistant’ colonies, Fig. 1c). In contrast, some colonies that were severely bleached in May showed visual signs of symbiont recovery by August and full recovery by October 2019 (‘recovered’ colonies, Fig. 1d). These two types of colonies (resistant vs. recovered) provide a natural experiment to better understand the reproductive consequences of bleaching in *Acropora hyacinthus* colonies showing different heat stress responses. Specifically, we postulated that (a) resistant colonies would have higher stored energy reserves than colonies that bleached and later recovered, (b) resistant colonies would be more likely to harbor developing gametes, (c) oocyte production would be more negatively impacted in colonies with prior bleaching, and (d) resistant colonies would produce more oocytes per polyp than recovered colonies. Mo’orean reefs have a history of recovery from disturbance: while coral reef community recovery is a function of multiple processes including coral recruitment, growth, survival of recruits, and regrowth of surviving colonies^34^, an important step in assessing possible resilience is examining reproductive potential (i.e., the ability of a colony to generate reproductive output) of surviving colonies, which we consider in this study.

## Results

### Energetic condition of resistant and recovered corals differed after recovery

**Figure 2:**
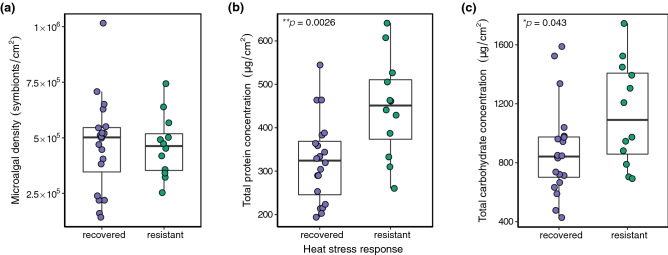
Endosymbiont density and energetic condition of recovered and resilient corals five months after the mass bleaching event. (**a**) Microalgal counts normalized to host surface area. (**b**) Total protein content normalized to host tissue surface area. (**c**) Total carbohydrate content normalized to host tissue surface area. Each data point represents a single colony.

Five months after the peak of the thermal anomaly and bleaching event, *Acropora hyacinthus* colonies in the field were categorized as dead, recovered, or resistant to bleaching (Fig. 1, Supplementary Table S1). Endosymbiont density did not differ significantly between resistant and recovered colonies (*p* = 0.96; Fig. 2a), which were visually healthy (Fig. 1c,d). Energetic condition was assayed in these colonies; total protein concentration and total carbohydrate concentration differed significantly between resistant and recovered corals. Resistant colonies had, on average, 123.09 µg/cm^2^ higher protein concentrations compared to resilient corals (46.55–199.63, 95% CI, *p* = 0.0026; Fig. 2b). They also had 250.48 µg/cm^2^ higher carbohydrate concentrations (8.07–492.90, 95% CI, *p* = 0.043; Fig. 2c).

### Bleaching resistant corals were more likely to harbor mature gametes compared to recovered corals

**Figure 3:**
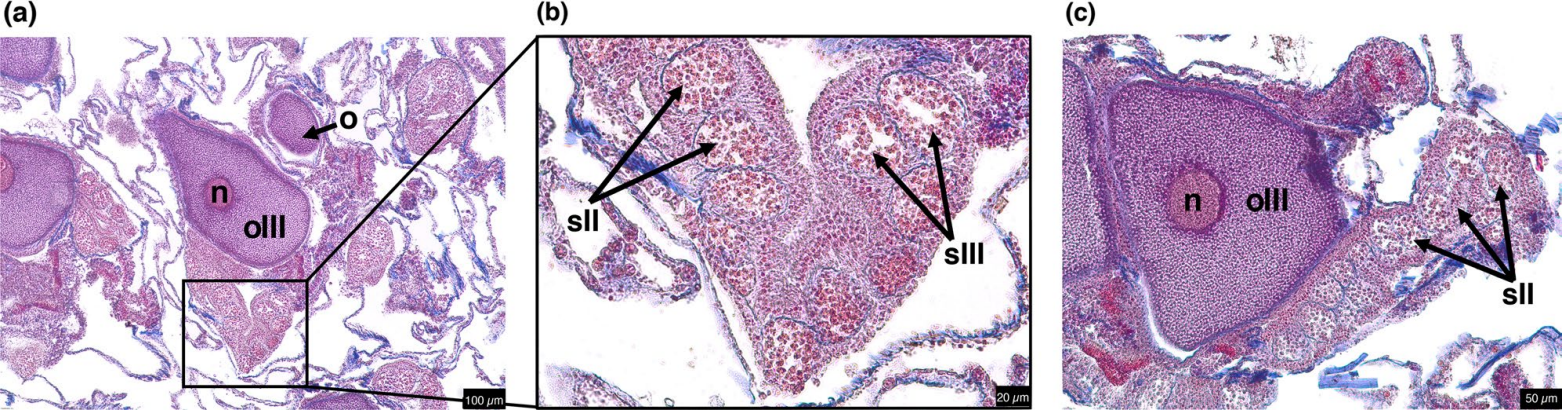
Histological sections of gravid *A. hyacinthus*. Scale bar represents (**a**) 100 µm, (**b**) 20 µm, and (**c**) 50 µm, respectively. *n* nucleus, *o* oocyte, *oIII* oocyte stage III, *sII* spermary stage II, *sIII* spermary stage III.

**Figure 4:**
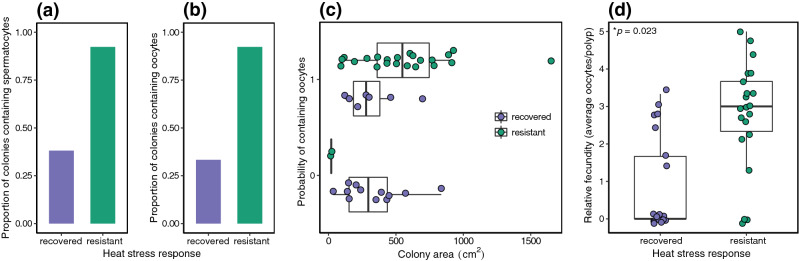
Differences in reproductive output between resistant and recovered coral colonies. Proportion of recovered and resistant colonies containing (**a**) spermatocytes and (**b**) oocytes. (**c**) Probability of containing oocytes for recovered and resistant colonies over the range of colony area. 0 means a colony did not contain oocytes. 1 means a colony contained oocytes. (**d**) Relative fecundity in recovered and resistant colonies. Each data point represents one colony.

To investigate long-term impacts on fitness between the two heat stress responses, we performed reproductive histology on colonies collected in October 2019 (Fig. 3), which falls within the typical spawning season for *Acropora hyacinthus*^35^*.* We observed a strong difference in reproductive potential between resistant and recovered coral colonies (Fig. 4). A total of 24 out of 26 (92.31%) resistant colonies contained gametes, while only 8 out of 21 (38.10%) recovered colonies contained gametes. Spermatocytes were observed in 24 out of 26 (92.31%) resistant colonies, but only 8 out of 21 (38.10%) recovered colonies (Fig. 4a). Oocytes were observed in 24 out of 26 (92.31%) resistant colonies and in 7 out of 21 (33.33%) recovered colonies (Fig. 4b). The probability of a colony containing spermatocytes or oocytes varied significantly with heat stress response. Resistant colonies were 36.00 times more likely to contain both spermatocytes and oocytes compared to recovered corals (3.13–978.89, 95% CI, *p* = 0.0089; Fig. 4c).

### Gamete stage and oocyte diameter did not differ between resistant and recovered corals

Among colonies containing spermatocytes, there was no significant difference in observed spermatocyte stages between recovered and resistant corals (*p* = 0.84). The majority of spermatocytes (686/723, 94.88%) were documented in stage II, with resistant and recovered colonies displaying 98.28% (514/523) and 86% (172/200) of spermatocytes in stage II. The remaining spermatocytes were observed to be in stage III.

Among colonies containing oocytes, there was no significant difference in oocyte size between resistant and recovered corals (*p* = 0.81; Supplementary Fig. S1). The average oocyte diameter for resistant colonies (N = 437) was 312.03 µm, and 309.75 µm for recovered colonies (N = 94). There was no significant difference in oocyte stages between the two heat stress responses (*p* = 0.99). The majority of oocytes (525/537, 97.77%) were observed in stage III, with resistant and recovered colonies displaying 97.75% (434/444) and 97.85% (91/93) of oocytes in stage III, respectively. The remaining oocytes were observed to be in stage IV.

### Relative fecundity was higher in resistant corals

To further understand the influence of the observed heat stress responses on fitness, we measured relative fecundity for each colony. On average, resistant colonies exhibited a relative fecundity of 2.78 (± 1.45, SD) oocytes per polyp, while recovered colonies exhibited a much lower relative fecundity of only 0.81 (± 1.24, SD) oocytes per polyp. Resistant corals produced 4.17 times as many oocytes compared to recovered corals (1.47–18.94, 95% CI, *p* = 0.023, Fig. 4d). Although colony area was associated with higher relative fecundity, this overall trend was not significant (*p* = 0.11, Supplementary Fig. S2). For resistant colonies, colony area had a marginally significant positive correlation with relative fecundity (r^2^ = 0.18, *p* = 0.052). For recovered colonies, colony area and fecundity showed no significant relationship (r^2^ = 9.43 × 10^–6^, *p* = 0.99).

## Discussion

Energy reserves represent an important currency for physiological performance, including processes such as growth, maintenance, and reproduction, all of which are tightly intertwined with organism survival and fitness^18^. Following severe stress events, individuals may allocate these energy reserves towards recovery rather than reproduction, delaying population recovery after disturbances with high mortality^8,36^. Here, we examined the energetic condition and reproductive effort in *Acropora hyacinthus* individuals showing heat stress responses of resistance and recovery. Despite both responses maintaining healthy appearances and high endosymbiont densities five months post-bleaching, our findings reveal divergent physiological responses that were not visually detectable during the October field surveys. Bleaching stress also resulted in compounding impacts on reproduction in recovered colonies. Not only were recovered colonies less likely to contain gametes than their resistant counterparts, but they also exhibited lower fecundity per polyp. Taken together, our study suggests that bleaching imposes an energetic constraint on both stress recovery and gamete production in *A. hyacinthus*, reflecting energy allocation strategies among processes, which may hamper reef recovery from disturbance.

Following the 2019 bleaching event in Mo’orea, visual colony recovery was a poor indicator of the coral animal’s energetic state. This is consistent with previously published studies which show quick, short-term recovery of photopigmentation or endosymbiont concentrations, but much longer recovery times for host energy reserves^14,17,37^. For example, an experimental bleaching study in *Porites compressa* found that although healthy pigmentation returned within 1.5 months of recovery, energy reserves (i.e., protein, carbohydrate, and lipid concentrations) remained depressed until 8 months of recovery^14^. We observed complete visual and endosymbiont recovery of bleached colonies within five months of the bleaching event; however, their energy reserves were still depleted. Protein and carbohydrate levels were 27.5% and 22.1% lower, respectively, than resistant colonies. This indicates that previously bleached colonies are catabolizing protein and carbohydrate reserves during or after the bleaching event, and demonstrates that lipid reserves are not the sole metabolite pool drawn from during stress recovery, as is commonly assumed for corals^12,38^. Although endosymbiont populations had evidently recolonized the coral tissues following bleaching, the translocation of photosynthates from symbiont to host may not have been fully restored^39^, forcing the coral to catabolize energy reserves to fulfill its metabolic requirements. Past studies have shown that it can take more than 11 months of recovery to reestablish pre-bleaching levels of nutrient transfer from symbionts to the coral host and to replenish host energy reserves^17,40^. It is currently unknown how long it takes *A. hyacinthus* to accumulate lost energy reserves after bleaching, but our study clearly shows that it requires greater than five months. With the threat of annual bleaching looming in the future^3^, failure to fully recover energy reserves within a year could compromise corals’ ability to effectively cope with further stressors^6,41^, leading to reduced reproductive output^20,21,23^.

Energy reserves, particularly lipids, play an essential role in determining coral reproductive output and larval survival^20,42^. Lipids compose up to 86% of oocyte biomass in broadcast spawners^26^, such as *A. hyacinthus*. During gametogenesis, stored energy reserves are provisioned to the developing gametes^24,43^ and they serve as the primary energy source for planulae during dispersal^44^. The gametogenic cycle for *A. hyacinthus* lasts for approximately nine months. Oogenesis initiates about six months prior to spermatogenesis and both male and female gametes reach maturity synchronically^45^. Spawning for this species in Mo’orea typically occurs in October to November^35^, meaning that oogenesis for the colonies in our study likely initiated around January 2019, one month after the onset of the thermal anomaly and four months before bleaching was first observed in April. Thus, corals underwent much of gametogenesis under thermal stress, with the bleaching event starting around month four of oogenesis and two months before the beginning of spermatogenesis. Because energy reserves are consumed as a recovery response to bleaching stress^17^, this incurs a significant cost to resources that would normally be allocated to gametogenesis^29,43^, which could explain the decrease in fecundity and gamete production we observed in colonies that bleached but later recovered. The importance of energy reserves for oocyte production is well-known^25,46,47^, but no such link has been established for sperm in corals. Our study provides evidence that depleted energy reserves may hinder both oogenesis and spermatogenesis, a process generally relegated as energetically inexpensive.

Oocyte size is a common metric used to assess the quality of reproductive output because of its positive relationship with fertilization success, postzygotic survival, and maternal investment^48,49^. Oocyte size is an indicator of maternal condition; individuals with larger energy reserves are able to provision more proteins and lipids to their oocytes and thus produce larger oocyte sizes^43,49^. A decrease in oocyte size is often observed as a consequence of severe stresses such as coral bleaching^21,23,30^, yet we found remarkably similar distributions and averages of oocyte sizes between resistant and recovered colonies, despite clear differences in gamete presence. A study of *Acropora millepora* demonstrated that under thermal stress, colonies maintained oocyte sizes across bleaching phenotypes, but produced fewer oocytes compared to when they reproduced under non-bleached conditions^25^. Because oocyte size is correlated with maternally provisioned lipids, this response is hypothesized to ensure adequate energy is available for all of the now limited number of oocytes to survive through settlement^25^. Since we observe a similar phenomenon to this study, it is possible that the strategy of producing fewer high quality oocytes over more low quality oocytes is specific to *Acropora,* as opposed to other coral genera where this strategy has not been observed^21,23,30^. In *A. hyacinthus,* bleaching stress appears to induce energetic constraints on reproduction, resulting in hosts provisioning a baseline level of nutrients into oocytes, with the number of oocytes being limited by the energetic costs of recovery. Oocyte size estimates in this study were notably smaller than in some previous studies^50,51^, which is likely attributable to our methodology, as histological processing is known to cause coral oocyte tissue to shrink up to 30% in comparison to dissected oocytes^52^. Furthermore, we collected samples in October, the beginning of the spawning season^35^, where we observed most oocytes in stage III, indicative that they had not yet reached their mature size^53^. However, we cannot definitively rule out bleaching-induced discrepancies in oocyte size. Future in situ surveys of *A. hyacinthus* reproductive traits will help elucidate the drivers of oocyte size observed during the 2019 bleaching event.

Colony size influences both bleaching susceptibility and fecundity^54–56^, but the size-dependent effects of bleaching on reproductive output are only recently coming to light^23^. In contrast to previous research which demonstrated that larger colonies were less likely to have reduced reproductive fitness following bleaching^23^, we found that colony size was not a significant factor in determining reproductive output in recovered and resistant colonies. For resistant colonies, there was a marginally significant effect of increasing polyp fecundity with increasing colony size, as expected in corals^57^, but this relationship was not present for recovered colonies, likely because the majority of polyps measured contained no oocytes, regardless of colony area. Coral reproductive maturity depends on both colony age and size^58^. *Acropora hyacinthus* reaches reproductive maturity around four to five years of age, which corresponds to a minimum colony diameter of about 7 cm^45^. All colonies we measured were larger than this threshold, except for two (one recovered and one resistant): these may not have been reproductively mature at the time of sampling, as neither were observed to contain gametes. However, colonies lacking oocytes were not limited to small, and thus possibly immature, colonies, and virtually all colonies that produced no oocytes underwent bleaching. Therefore, the trends in reproductive output we observed likely represent a true biological signature of bleaching, not an artifact of colony size or age. Additionally, we documented substantial overlap in colony size range between the resistant and recovered colonies, but none of the recovered corals were very large colonies (> 1000 cm^2^). During the 2019 bleaching event, mortality for *Acropora* colonies was size-dependent and larger individuals (≥ 30 cm diameter) were more likely to die as a result of bleaching stress^5,33,59,60^. Large colonies contribute a disproportionate amount of reproductive material compared to small colonies due to their higher per polyp fecundity and larger surface area^54,57^. Thus, the loss of large colonies has serious consequences for the overall population reproductive output, reducing recruitment and delaying coral community recovery^61^.

Intraspecific bleaching severity and recovery can vary across different habitats and are shaped by environmental factors such as light intensity, water flow, and water temperature^62–64^. We recorded local-scale heterogeneity in heat stress response, partitioned by depth. Coral communities at the shallower (~ 5 m) and deeper (~ 14 m) depths both experienced bleaching during the 2019 thermal anomaly, but bleaching and subsequent mortality of *A. hyacinthus* were much more extensive at deeper depths. Furthermore, colonies resistant to bleaching were only observed at the shallower depths, while recovered colonies were seen at both depths. The spatially variable bleaching patterns we documented are consistent with previous studies^65,66^; for example, differential bleaching susceptibilities associated with depth were reported in Mo’orea during the 1994, 2002, and 2007 bleaching events, with coral assemblages displaying less severe bleaching at 6 m depth than at 12 m and 18 m^67,68^. Together, our observations indicate a higher thermotolerance in corals from shallower depths at this location. Both depths likely experienced similar heat stress exposures during the bleaching event, which suggests that the variation in bleaching response is, at least in part, driven by local environmental conditions, particularly higher light intensity and/or greater daily temperature fluctuations at shallow depths^64,69^. These differences in habitat microenvironments may have conditioned colonies at ~ 5 m to be more robust to extreme heat stress than deeper colonies through long-term acclimatization or local adaptation^38,70^. However, we acknowledge that our study only examined one reef during the 2019 bleaching event and depth-associated bleaching patterns may have varied across the island. Avoiding the energetic cost of bleaching allows resistant corals to provide critical gamete stocks for stress-tolerant populations, promoting multigenerational resilience to a rapidly changing climate.

Temperature exerts a strong influence on coral gametogenic and spawning cycles^53^. Recent thermal events have underscored the fact that bleaching and heat stress can depress coral reproduction. For example, the 2015 bleaching event in Hawai’i was followed by a reduction in spermary and oocyte production for multiple reproductive seasons in *Pocillopora meandrina*^23^. On the Great Barrier Reef, bleaching in 2016 and 2017 resulted in an 89% decline in recruitment^61^. Similar patterns have been documented elsewhere, including the Persian Gulf, where consecutive mass bleaching preceded a 58% decrease in settlement^71^. Bleaching stress acts on populations by first removing individuals due to mortality and then impeding reproductive success in survivors through reductions in oocyte size, fecundity, and settlement. Because the success of surviving colonies can influence reef recovery trajectories, understanding the reproductive and energetic ramifications of bleaching on surviving colonies is crucial. Our study adds to the growing body of literature demonstrating the deleterious impacts of thermal stress and bleaching on coral physiology and reproduction, and is the first, to our knowledge, to utilize reproductive histology to investigate these questions in *A. hyacinthus*. We identified *A. hyacinthus* colonies displaying one of two distinct temperature stress responses and, by combining analyses at the cell, polyp, colony, and site levels, we confirm that bleaching impairs reproduction and is related to the energetic state of the coral during the reproductive season immediately following the 2019 mass bleaching event in Mo’orea. Both phenotypes appeared visually recovered within five months after bleaching, but previously bleached colonies harbored diminished energy reserves coupled with significantly reduced gamete production and fecundity compared to colonies with no history of bleaching. Our results emphasize the importance of considering the invisible, sublethal effects of thermal anomalies in assessing reef health. Further, we likely underestimate the consequences of this bleaching event on coral reproduction since we did not evaluate other potential impacts such as perturbed spawning synchrony^72^, larval mortality^73^, or suppressed settlement and recruitment^61,71^. Because these processes can be highly variable on spatiotemporal scales^34,74,75^ and our study focused on a single reef, assessing the possibility of hampered reef recovery dynamics as a result of bleaching will require extensive data collection in addition to the data presented herein. Corals exhibit intraspecific variation in response to extreme thermal stress, as we show, which can be harnessed for investigating community resilience dynamics. As coral bleaching and other anthropogenic disturbances increase in magnitude and frequency^2^, more than ever there is a critical need to understand inter- and intraspecific variation in recovery and reproductive success.

## Methods

### Study site and sampling scheme during and after mass bleaching in situ

Mo’orea, French Polynesia is a volcanic island with a ~ 60 km perimeter in the South Pacific Ocean. The island is surrounded by a barrier reef system with lagoons up to 1.3 km in width, surrounded by a reef crest and forereef habitat. The forereef, where we conducted our study, maintained ~ 47% live coral cover (mean of six outer reef sites at 10 m depth) as of January 2019^76^. We conducted our study at one site located on the north shore of Mo’orea (17.4731° S, 149.8177° W; Supplementary Fig. S3). Individual *Acropora hyacinthus* coral colonies were observed, photographed, and tagged between 5 and 14 m depth on the forereef in May 2019, at the height of the mass bleaching event. At ~ 14 m, 59/111 tagged colonies were observed in a severe state of bleaching and 52/111 were partially bleached, while at ~ 5 m 16/52 colonies were severely bleached, with the remaining 36/52 maintaining a visibly healthy state. By August 2019, all the previously tagged colonies at ~ 14 m depth had died. Although mortality at ~ 14 m was high, in August, 25 additional (previously untagged) colonies were observed to be visibly recovering from bleaching (Fig. 1b). These recovering colonies were photographed and individually tagged. We note that survey timing during extended thermal stress events, such as the one investigated here, can influence perceptions of coral susceptibility to bleaching^77^. However, because of the high prevalence of bleaching at this site and depth during May (53.2% severely bleached and 46.8% partially bleached), we are confident that these previously untagged colonies were bleached during the bleaching event. In October 2019, 30 and 28 previously tagged colonies at ~ 5 and 14 m, respectively, were found, photographed, and sampled via SCUBA for physiological metrics and/or reproductive histology (see Supplementary Table S1 for full sample details). For all corals sampled, bleaching severity and colony area were determined using standardized photographs. Each colony was assigned a score from 1 to 5 according to the bleaching severity the colony experienced, with a 1 indicating stark white bleaching and a 5 indicating no visible bleaching (Fig. 1b). Colony area was estimated by tracing the outline of each colony and calculating the planar surface area using ImageJ^78^.

Water temperature data (Fig. 1a) were collected as part of the Mo’orea Coral Reef Long Term Ecological Research (MCR LTER) time series data collection^79^. Data were collected at six MCR LTER sites at 10 m depth on the forereef using bottom-mounted thermistors (Seabird SBE 39) that recorded the water temperature every 20 min. To evaluate long-term temperature trends on the outer reef we considered data through October 31, 2018. We first calculated the average temperature at each site for each day of the time series. We then used the daily site average to calculate the average water temperature across all sites ± one standard deviation for each day in a 365-day year. We used the same approach to calculate the average daily temperature for late 2018–2019 using data from November 1, 2018–October 31, 2019.

### Physiological condition of corals following mass bleaching

In tagged coral colonies that were resistant to or recovered from bleaching, the energetic condition of the host was assessed (N_resistant_ = 12, N_recovered_ = 20). One small branch (~ 2–4 cm length) was sampled from each colony and airbrushed in filtered seawater to remove coral tissue and algal cells (blastate) from the skeleton. The blastate was homogenized and 200 µL was collected and preserved in Z-fix (10% zinc formalin) for algal symbiont counts. The remaining blastate was centrifuged at 2000×*g* for 2 min to separate the host tissue from endosymbiont cells. Host tissue slurry was preserved at − 20 °C until further processing. Microalgal endosymbiont density was quantified using a hemocytometer (Hausser Scientific, Horsham, PA) under an Olympus BH-2 microscope. Total host protein content was quantified using a Bradford assay^80^ with bovine-serum albumin (BSA) as a standard (Pierce Coomassie Plus Assay Kit, Thermo Fisher Scientific). Briefly, host tissue homogenate was diluted 10 × and triplicate 100 µL aliquots were loaded onto a 96-well plate and mixed with 200 µL of Bradford reagent. After a 10-min incubation period at room temperature, absorbance at 595 nm was recorded with a microplate reader (BioTek PowerWave XS). Sample protein concentrations were calculated using a standard curve of BSA ranging from 0 to 120 µg/mL. Total host carbohydrate content was quantified using a modified phenol–sulfuric acid method^81,82^. Triplicate 50 µL aliquots of host tissue homogenate were loaded onto a 96-well plate and mixed with 150 µL of concentrated sulfuric acid immediately followed by 30 µL of 5% phenol. Samples were incubated at 90 °C for 5 min and then allowed to cool. Absorbance was measured at 490 nm with a microplate reader and sample carbohydrate concentrations were determined using a standard curve of dextrose ranging from 0 to 3500 µg/mL. All physiological metrics were standardized to coral skeleton surface area following the paraffin wax-dipping technique^83^.

### Reproductive histology

Small fragments from tagged colonies were sampled by hand via SCUBA (N_resistant_ = 26, N_recovered_ = 21) in October 2019, the start of the typical spawning season for *A. hyacinthus*^35^. For each colony, the selected fragment was sampled 5–10 cm from the colony edge, and branch tips and colony edges were avoided. Samples were immediately preserved in Z-fix for 24 h and then stored in 100% ethanol until histological processing. Samples were decalcified with a 1% EDTA decalcifier solution for 48–72 h and stored in 70% ethanol until processing on a Leica ASP6025 tissue processer. Paraffinized tissue was embedded in wax blocks (Leica EG1150H embedding machine) and then allowed to cool in a freezer 24 h prior to sectioning. Blocks were serially sectioned at 5 µm thickness on a Leica RM2125RTS microtome every 300 µm, which corresponds to the average oocyte diameter. Sections were arranged on microscope slides and stained using a modified Heidenhain’s aniline blue stain on a Leica ST5020 multistainer.

Histological sections were analyzed for measurements of reproductive effort: (1) presence/absence of male and female gametes, (2) diameter of oocytes, and (3) relative fecundity, detailed below. Gametes (oocytes and spermatocytes) were staged from I-V following the classification of Szmant-Froelich et al.^84^. Slides were examined using an Olympus BX41 microscope with an Olympus SC180 camera attachment. Measurements were made using ImageJ^78^. Oocyte diameter was determined by averaging the longest and shortest axis of each oocyte. A total of 25 oocytes were measured from each colony. In fragments containing fewer than 25 oocytes, the maximum number of oocytes observed was used (Supplementary Table S1). Only oocytes with a visible nucleus were measured to ensure no oocytes were counted more than once and that the maximum diameter was measured.

Due to the small size of the fragments and polyps, as a proxy for fecundity, three polyps were randomly selected on the middle slide from each individual. When there was an even number of slides, the first of the two middle slides was used. Because only one slide from each individual was examined, there was no risk of double-counting oocytes, so the number of both nucleated and non-nucleated oocytes was counted in each of the randomly selected polyps. These counts were averaged to produce the average number of oocytes per polyp for each individual as a measure of relative fecundity. It should be noted that this relative estimate is lower than true fecundity.

### Statistical analyses

All statistical analyses were implemented in R (V. 4.0.3). To determine how symbiont density and protein and carbohydrate content were impacted by bleaching history, a categorical linear regression was used. Bleaching history was defined as being either ‘resistant’ or ‘recovered’. Bleaching history was highly collinear with depth. Although depth was non-significant in all models, we chose to include it to control for the effects of depth on heat stress response. A mixed-effects model was employed to examine whether oocyte diameter was influenced by bleaching history, with fixed effects of bleaching history and depth and a random effect of colony identity to account for repeated measures. A linear regression was performed to assess the relationship between colony area and relative fecundity for each heat stress response.

The remainder of statistical analyses on the histological measurements were performed using generalized linear models (GLMs). To determine if recovered and resistant colonies differed in displayed oocyte and spermatocyte stages, log-linear models with a Poisson distribution were used with gamete stage, bleaching history, and depth as fixed effects, an interaction effect between gamete stage and bleaching history, and a random effect of colony identity. Oocyte and spermatocyte stages were analyzed separately. To determine if bleaching history affected the probability a colony contained gametes, a logistic regression was used with the binomial response variable being whether the gamete of interest was observed in the colony. Depth was included as a fixed effect and the presence of oocytes and spermatocytes were analyzed separately. A Poisson regression was utilized to examine how relative fecundity differed between bleaching histories and depth. We also used a Poisson regression to determine the effect of bleaching history and colony size on relative fecundity. The interaction term between colony size and bleaching history was found to be non-significant and was removed from the model. All model outputs and results are listed in Supplementary Table S2.

## Supplementary Information

Supplementary Information.

Supplementary Tables.

## Data Availability

All datasets and code generated as part of this study are available on GitHub at the following link (https://github.com/sarahleinbach/hyacinthus_histo). Photographs are available from the corresponding author on reasonable request.
