# Supplementary material for: Energetic and reproductive costs of coral recovery in divergent bleaching responses
Source: Sci Rep. 2021 Dec 7;11:23546. doi: 10.1038/s41598-021-02807-w (PMC8651640; doi:10.1038/s41598-021-02807-w)
Supplement: Supplementary file 1 — Supplementary Information. [file 41598_2021_2807_MOESM1_ESM.pdf]

Supplementary Material to:

Energetic and reproductive costs of coral recovery in divergent bleaching responses

Sarah E. Leinbach, Kelly E. Speare, Ashley M. Rossin, Daniel M. Holstein, Marie E. Strader

Supplementary Figure S1.

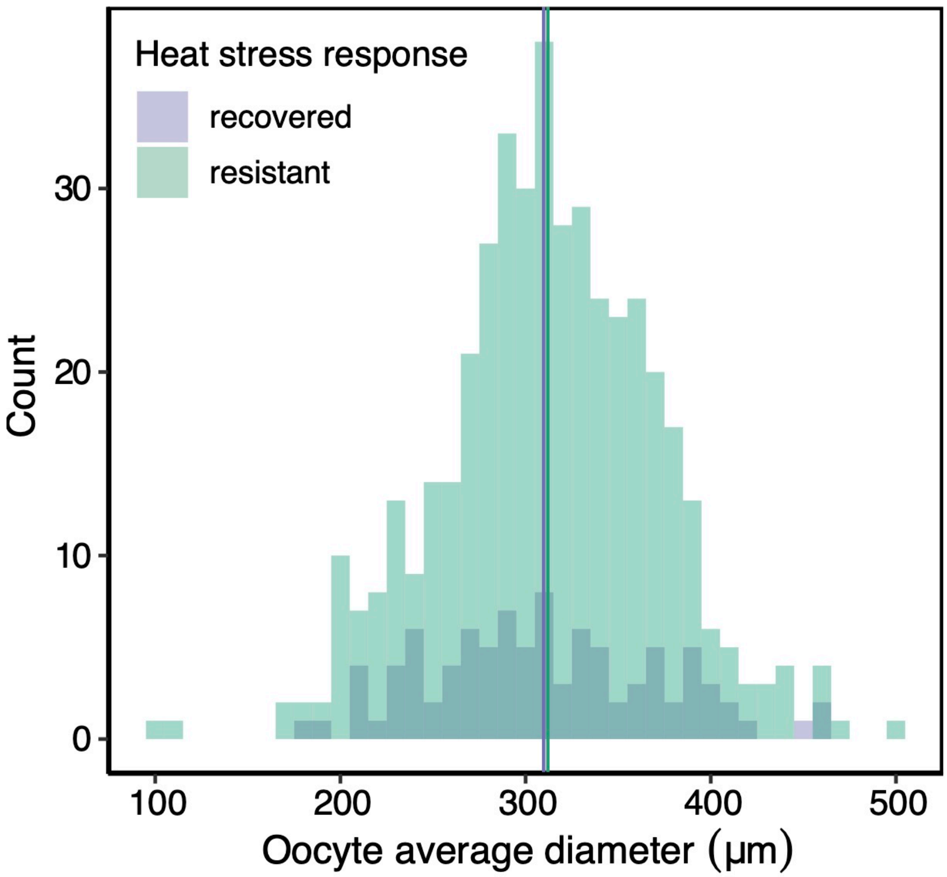

Supplementary Figure S1. Distribution of oocyte diameters in recovered (N = 94 oocytes) and resistant (N = 437 oocytes) colonies. Central vertical lines represent the average oocyte diameters.

Supplementary Figure S2.

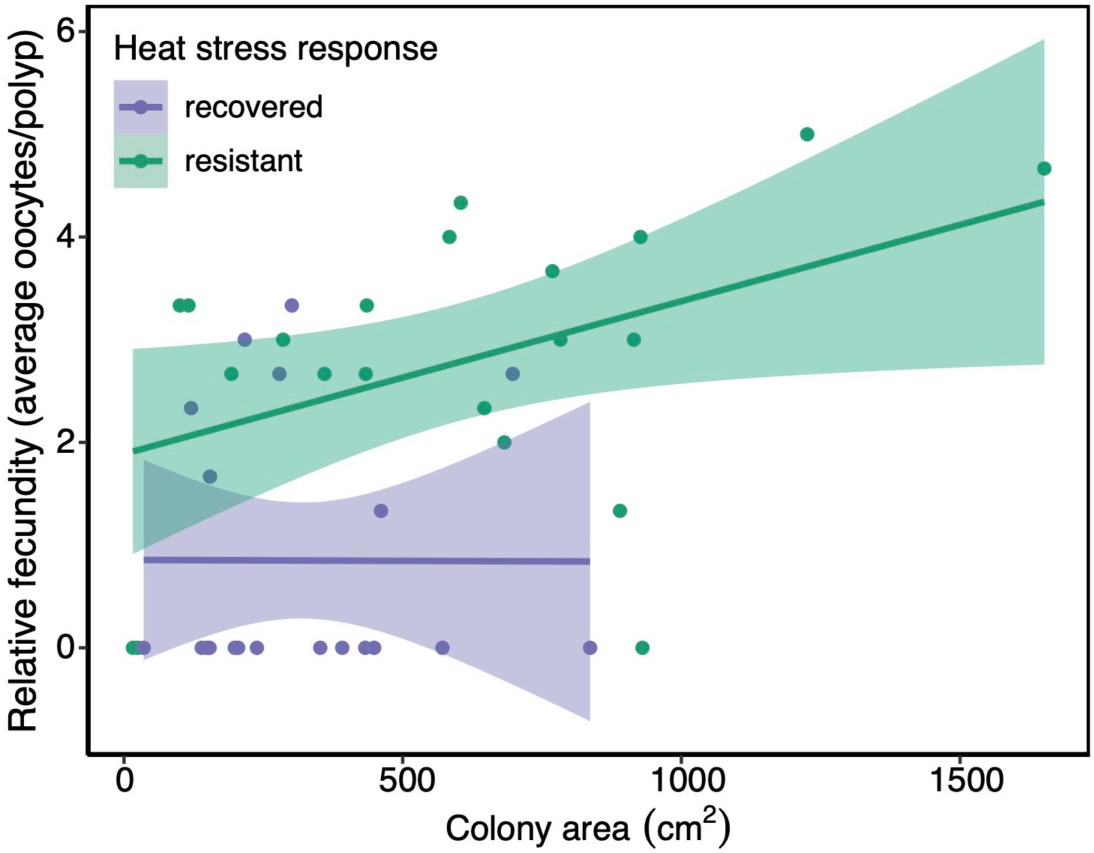

Supplementary Figure S2. Relative fecundity in recovered and resistant corals across colony size. Each point represents one colony. Shaded areas are 95% confidence intervals.

Supplementary Figure S3.

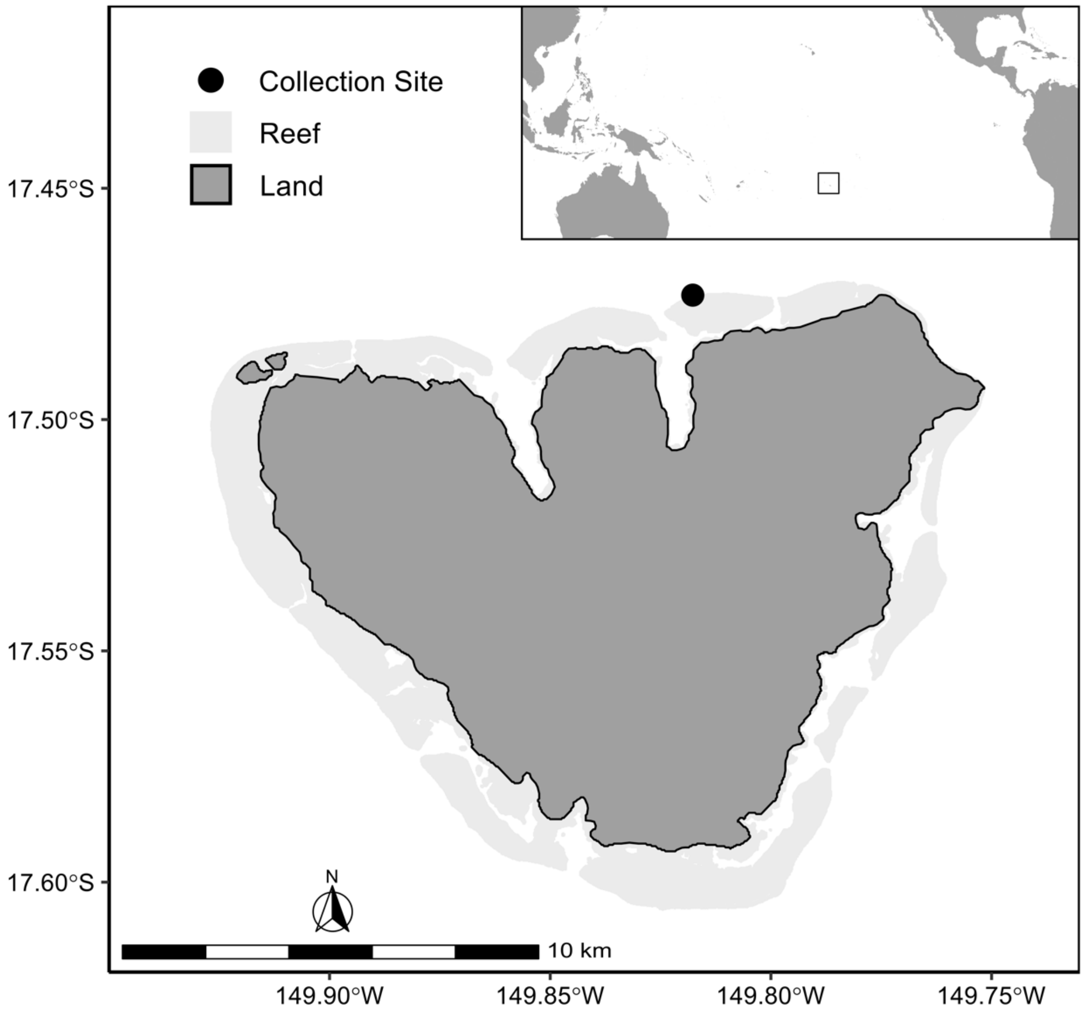

Supplementary Figure S3. Map of Mo'orea, French Polynesia showing the sampling site along the north shore (17.4731° S, 149.8177° W). Island shapefile adapted from OpenStreetMap[1]. Map data copyrighted OpenStreetMap contributors and available from https://www.openstreetmap.org.

1. OpenStreetMap contributors. Planet dump retrieved from https://www.openstreetmap.org (2015).

Supplementary Table S1.

Included as separate excel file. Spreadsheet detailing bleaching response and sampling details for all colonies in the study. Note that the majority of colonies were not sampled for both energetic and reproductive measurements. Cells filled with 'NA' means either the colony was not photographed or was not sampled for the particular metric of that column.

Supplementary Table S2.

Supplementary Table 2. Model results for all statistical tests. Results of: a) categorical linear regression testing the effect of heat stress response on physiological metrics; b) logistic regression testing whether heat stress response affected gamete presence; c) log-linear model testing for differences in gamete stage between resistant and recovered colonies; d) mixed-effects model testing the impact of heat stress response on oocyte size; e and f) Poisson regressions testing for differences in relative fecundity between heat stress responses. Asterisks denote $p < 0.05$ significance.

(a)

Test	Fixed factor	d.f.	t-value	*p*-value
Symbiont density	Heat stress response	30	-0.052	0.959
Protein content	Heat stress response	30	3.284	0.0026*
Carbohydrate content	Heat stress response	30	2.11	0.0433*

(b)

Test	Fixed factor	d.f.	z-value	*p*-value
Oocyte presence	Heat stress response	44	2.617	0.00887*
	Depth	44	0.390	0.69621
Spermatocyte presence	Heat stress response	44	2.617	0.00887*
	Depth	44	0.591	0.55454

(c)

Test	Fixed factor	z-value	*p*-value
Oocyte stage	Heat stress response	-0.013	0.98928
	Depth	-1.681	0.09270
Spermatocyte stage	Heat stress response	0.208	0.835
	Depth	0.000	1.000

(d)

Test	Fixed factor	t-value	*p*-value
Oocyte size	Heat stress response	-0.244539	0.8087
	Depth	-0.748876	0.4607

(e)

Test	Fixed factor	d.f.	z-value	*p*-value
Fecundity	Heat stress response	39	2.279	0.0227*
	Depth	39	0.352	0.7247
Fecundity over size	Heat stress response	38	3.535	0.000408*
	Colony size	38	1.606	0.108231

(f)

Test	Fixed factor	r^2	d.f.	t-value	*p*-value
Fecundity, resistant	Colony size	0.1849	19	2.076	0.05167
Fecundity, recovered	Colony size	9.43×10^{-6}	18	-0.013	0.9897
